# Application of machine learning to understand child marriage in India

**DOI:** 10.1016/j.ssmph.2020.100687

**Published:** 2020-12-05

**Authors:** Anita Raj, Nabamallika Dehingia, Abhishek Singh, Lotus McDougal, Julian McAuley

**Affiliations:** a Center on Gender Equity and Health, Department of Medicine, University of California San Diego, San Diego, CA, USA; b Department of Education Studies, Division of Social Sciences, University of California San Diego, San Diego, CA, USA; c International Institute of Population Sciences, Mumbai, India; d Department of Computer Science, School of Engineering, University of California San Diego, San Diego, CA, USA

**Keywords:** Child marriage, Machine learning, India

## Abstract

**Background:**

Prior research documents that India has the greatest number of girls married as minors of any nation in the world, increasing social and health risks for both these young wives and their children. While the prevalence of child marriage has declined in the nation, more work is needed to accelerate this decline and the negative consequences of the practice. Expanded targets for intervention require greater identification of these targets. Machine learning can offer insight into identification of novel factors associated with child marriage that can serve as targets for intervention.

**Methods:**

We applied machine learning methods to retrospective cross-sectional survey data from India on demographics and health, the nationally-representative National Family Health Survey, conducted in 2015–16. We analyzed data using a traditional regression model, with child marriage as the dependent variable, and 4000+ variables from the survey as the independent variables. We also used three commonly used machine learning algorithms– Least Absolute Shrinkage and Selection Operator (lasso) or L-1 regularized logistic regression models; L2 regularized logistic regression or ridge models; and neural network models. Finally, we developed and applied a novel and rigorous approach involving expert qualitative review and coding of variables generated from an iterative series of regularized models to assess thematically key variable groupings associated with child marriage.

**Findings:**

Analyses revealed that regularized logistic and neural network applications demonstrated better accuracy and lower error rates than traditional logistic regression, with a greater number of features and variables generated. Regularized models highlight higher fertility and contraception, longer duration of marriage, geographic, and socioeconomic vulnerabilities as key correlates; findings shown in prior research. However, our novel method involving expert qualitative coding of variables generated from iterative regularized models and resultant thematic generation offered clarity on variables not focused upon in prior research, specifically non-utilization of health system benefits related to nutrition for mothers and infants.

**Interpretation:**

Machine learning appears to be a valid means of identifying key correlates of child marriage in India and, via our innovative iterative thematic approach, can be useful to identify novel variables associated with this outcome. Findings related to low nutritional service uptake also demonstrate the need for more focus on public health outreach for nutritional programs tailored to this population.

## Highlights

• Machine learning (ML) finds higher fertility, geography, and low SES are associated with child marriage.
• ML findings are comparable to that of prior epidemiologic research.
• Developed methodology involving thematic analysis of ML expands capacities for hypothesis generation from ML approaches.
• Findings show poor use of public health programs on maternal and child nutrition to be related to child marriage in India.

## Introduction

Child marriage – or married prior to age 18 years – is a health and human rights violation affecting one in five girls globally, the majority of these in South Asia and sub-Saharan Africa (UNICEF, 2019; UNICEF. Global databases, 2019). Multinational studies document that child marriage is associated with increased risk for reproductive, maternal, and nutritional health risks, as well as marital violence, even as these young brides age into adulthood (Efevbera et al., 2019; Kidman, 2017; Raj, 2010; Santhya, 2011). Studies also demonstrate association between early marriage of mothers and poor infant and child health outcomes including preterm birth, malnutrition, and even mortality (Efevbera et al., 2017; Khan et al., 2019; Nasrullah et al., 2014; Rahman et al., 2018; Raj, 2010; Raj & Boehmer, 2013; Raj et al., 2010a).

This evidence on health correlates of child marriage, developed largely during the past decade as part of global investments and commitments to end child marriage, demonstrates the impact of this issue. Correspondingly, cross-national research on socio-demographic correlates of child marriage, including poverty, rural residence, and non-attendance in school, also demonstrate that this is an issue of development (Raj, 2010; UNICEF, 2018; UNGA, 2018). Resultant programmatic and policy work developed from this research, as well as intervention evaluation studies documenting the value of cost transfers and incentivization schemes to retain girls in school as well as life skills training for reduction in child marriage (Kalamar et al., 2016), have contributed to national investments toward multi-sectoral efforts in child marriage prevention (Lo Forte et al., 2019). With these evidence-based investments accelerating change, we have seen a 15% reduction in girl child marriage globally over the past decade, as well as improvements in girl school attendance and delayed age at first birth (UNICEF, 2019; UNICEF. Global databases, 2019).

While these improvements are laudable, research and policy experts have confirmed that the annual rate of reduction in prevalence of child marriage – currently at 1.9% - would need to increase by 23% in order to achieve the Sustainable Development Goal (SDG) target 5.3 to end child marriage by 2030 (Lo Forte et al., 2019). In the absence of this improvement in acceleration, UNICEF estimates that more than 150 million more girls will marry prior to age 18 years by 2030 (UNICEF, 2018). Accordingly, the UN General Assembly in October 2018 renewed their commitment to eliminate child marriage by 2030, and called for an increased recognition to move beyond focus on structural factors related to child marriage – specifically poverty and education (UNGA, 2018). They also recommend greater emphasis on the role of social norms in maintaining the practice, often reinforced by families and communities in ways that undermine girls' agency and gender equality goals more broadly (UNGA, 2018). As with the prior efforts at acceleration building on the evidence base, so too does this call align with the growing body of research highlighting social norms related to age at marriage, female engagement in marital choice, and expectations of women and girls’ roles in education, work and society in affecting child marriage practices (Cislaghi et al., 2019a, 2019b; Kenny et al., 2019; Steinhaus et al., 2019; Taylor et al., 2019). Advancements in theory and science of social norms affecting traditional practices have been instrumental in these considerations.

Overall, the use of research evidence appears to offer much value in improving understanding of, and approaches to addressing child marriage. But, as we advance our intervention and policy approaches building on this evidence, we must also consider advancing our research methodologies to guide innovative hypothesis generation in the area. Our current methods in this field rely almost exclusively on traditional epidemiologic and demographic analysis of large scale nationally representative survey data available from child marriage affected nations, where we a priori select potential correlates for consideration, and test our hypotheses of expected correlations. To that end, approaches from machine learning offer promise in their capacity to offer a hypothesis generating approach, in ways that can expand public health research methodologies, particularly for exploration of novel variables (Bellinger et al., 2017; Mooney & Pejaver, 2018a).

Machine learning is a vast and rapidly expanding field, and at its most basic is the practice of using algorithms to parse data, and learn from it, with an objective of uncovering relationships among the variables either for predicting, classifying, or simply identifying patterns in the data. [Note: Machine learning language uses the term “predict” in assessment of correlations; our data are cross-sectional, precluding assumptions of causality implied by the term “predict.” For the purposes of this paper, the “prediction” is actually a test of association.] The machine itself runs through multiple iterations and learns the optimum model based on the type of task and algorithm chosen (Bellinger et al., 2017; LeCun et al., 2015; Mooney & Pejaver, 2018a). One of the key ways in which machine learning can differ from the traditional epidemiologic methods is by being oriented toward hypothesis generation rather than hypothesis testing (Bellinger et al., 2017). Machine learning algorithms can be applied for mining large scale datasets, allowing us to consider a range of variables available in our large survey data sets that we might not otherwise hypothesize to be related to our given outcome of interest. This association may be for spurious reasons or because the theoretical underpinnings guiding our hypotheses require expansion. Although, by using specific algorithms suitable for large datasets with potentially large number of variables that might be unrelated to the outcome of interest, we can mitigate the identification of such noisy relationships. Machine learning algorithms can thus support hypothesis generation (Mooney and Pejaver, 2018b), much the way qualitative research might (Glaser & Strauss, 1999), requiring both domain as well as methodologic expertise to interpret findings for hypothesis generation and theory expansion. While still relatively new in terms of use in public health, we can find its application in the areas of environmental health, physical health, cognitive health, and even social determinants of health (Bellinger et al., 2017; Bratic et al., 2018; Daoud et al., 2019; DeGregory et al., 2018; Seligman et al., 2018). We have found no evidence of its application to the topic of interest for this study, child marriage.

In this study, we use machine learning to identify variables related to child marriage based on survey data from a very large nationally representative sample of women aged 20–24 years in India. We focus on these data because of the expansive analyses on the topic of child marriage conducted using this surveillance data in prior years of study, including work conducted by authors of this work. Extensive prior research on the topic allows us strong domain and survey data understanding (Raj et al., 2009, 2010a). Additionally, this data set is both “wide” (with a large number of variables) and “tall” (with a large number of subjects), making machine learning beneficial to maximize use of all possible variables, and identify potential relationships among variables that have not previously been considered. Further, India continues to have the largest number of girls married prior to age 18 of any nation in the world, and at the same time demonstrates a substantial drop in the practice from 2005-06 [47%] to 2015-16 [27%] (IIPS, 2018). Hence, exploration of this topic with this India data set allows for identification of unconsidered and unexplored risk factors that we can target to accelerate change should a “ceiling effect” take root in the observed reduction of the practice. Analysis of these data will not only provide insight into variables or constructs related to child marriage that are heretofore not well understood or targeted by current efforts, this analysis can also provide insight into the validity of machine learning to identify strong correlates of child marriage, given the extensive prior research on this topic using traditional analyses.

## Materials and methods

We used data from the National Family Health Survey (NFHS-4), a nationally representative Indian household survey (IIPS, 2018). The survey was conducted from 2015 to 2016, and interviewed women of age 15–49 years. It included questions on socio-demographic characteristics of the respondent, her marriage and co-habitation history, contraception use, family planning services, contacts with community health workers, pregnancy, delivery, postnatal care and children's nutrition, fertility preferences of the woman and other health issues. This analysis includes the women who reported to be ever-married, and were 20–24 years of age (N = 78,542), to allow for analysis of child marriage as more recently practiced. The international standard for prevalence estimates of child marriage are based on prevalence of its occurrence among 20–24 year olds, because this age offers sufficient time for a marriage to have occurred, while at the same time offering a population young enough to reflect on more current marital practices.

### Measures

Our primary outcome was child marriage, as reported by the woman. We categorized those who reported marriage before the age of 18 as cases of child marriage.

We adopted an exploratory approach for this study, with the intention of discovering novel predictors that might be associated with child marriage among women of age 20–24 years. Variables from all sections of the NFHS-4 survey were thus included in our statistical models. However, before implementing the statistical models, the data variables were pre-processed in order to ensure interpretability of results from the machine learning algorithms. First, two domain experts on the team familiar with the NFHS-4 survey data reviewed each variable in the dataset, and a) eliminated certain variables that were not meaningful for analysis, for example variables related to date of interview, case ID, and village ID, and b) identified the different categories each variable should include. These were based on the categorizations for variables used in prior research, which would allow us to interpret our findings in a manner consistent with existing literature. The two reviewers discussed and resolved any disagreements related to categorizations. Once the variable categories were identified, we one-hot encoded the categorical variables and normalized the continuous variables. One hot encoding is a process by which categorical variables are converted into multiple binary forms. In total, each woman was represented as a set of over 6500 features or variables, that summarized their personal as well as health status.[As we describe analyses, we use the term features rather than variables because each variable has been deconstructed, or one hot encoded into multiple dummy variables, with each dummy variable constituting a feature.] While one-hot encoding allows us to draw meaningful results from the machine learning models, we do note one of its limitations that it increases the overall number of features in our analysis (Johannemann et al., 2019). However, given the specific objective of our study, we prioritize the interpretation of results over a reduction in the number of variables.

### Statistical analysis

In this study, we use supervised machine learning models, which are predictive models; they learn, or identify the best model, from a given set of data where the outcomes are labelled, i.e., they are already categorized as positive and negative. The identified best-fit model is then used to predict outcomes in another labelled dataset, known as test dataset. For any machine learning task, the data set is thus split into: a training dataset which is used to train the model, a test dataset where we predict the outcomes and check if the predicted outcomes are similar to the actual outcomes, and a validation dataset which is used to estimate the parameters to be included in the training models. We randomly assigned 20% of the sample to our test dataset. For the remaining 80 percent, we used k-fold cross-validation, a method of assessing how well a model can be generalized to an independent data set. In this method, the data are partitioned into k subsets of approximately equal size and one of the subsets becomes the validation set. The remaining k-1 subsets are used as training data. All models were first ran or trained on the training dataset. We estimated the metrics for evaluation of the model's accuracy and error rates using the test dataset. We compared three distinct different strategies, to provide greater insight into what features are meaningful across approaches: traditional logistic regression, lasso and ridge regression, and lasso and neural networks. These models were selected based on their longstanding and continued popularity in machine learning for classification tasks (Muthukrishnan & Rohini, 2016; Ng, 2004; Stock et al., 2012) and their recognized and recommended use in public health and behavioral science research (Ghaoui et al., 2010; McNeish, 2015; Mooney and Pejaver, 2018b; Seligman et al., 2018). Analysis of the same data set using three machine learning approaches also allow for more robust consideration of findings. We conducted all analyses using Python with necessary libraries (pandas, scipy, keras, numpy, sklearn, tensorflow) to develop the predictive algorithms. [Code available upon request from authors.]

Traditional Logistic Regression. We first used a traditional logistic regression model, with child marriage as the dependent variable, all the selected variables as the independent variables (i.e., features for prediction), and no cross-validation. This approach differs from standard epidemiologic analyses using logistic regression, as epidemiologic analyses select predictor variables for a given outcome a priori, based on hypotheses. Traditional logistic regression in machine learning, as noted previously, allows for consideration of all possible variables in a data set as predictors, allowing the machine rather than the researcher to determine what features or variables predict the outcome. However, in datasets with a large number of features, the traditional logistic regression is vulnerable to multicollinearity and over-fitting. This is also the case in hypothesis driven regressions used in epidemiologic research, but it can be addressed via a process of model development prior to testing the model, which is not the case for machine learning.

**Fig. 1:**
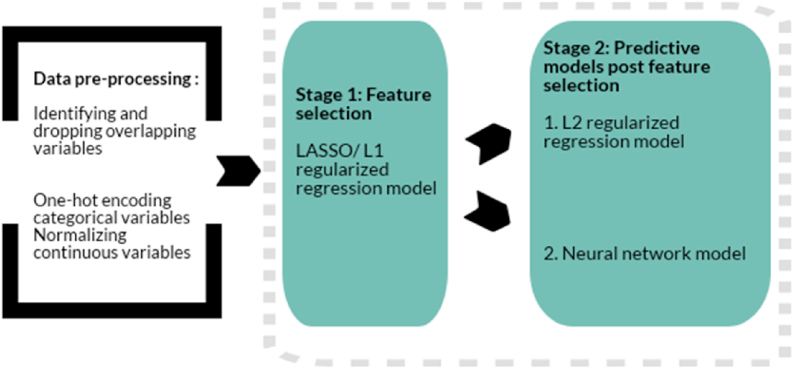
Approach to identifying predictors.

Lasso and Ridge Regression. Second, we used regularized lasso and ridge models. While there are multiple machine learning algorithms available that can be used for classification tasks, the decision to implement these two specific models was based on the specific context of our research question, and discussions among gender research and computer science experts on the team. Our dataset in this study has a large number of features (~6500, after one hot encoding), which can hinder correct parameter estimation when trying to build a traditional logistic regression model. We address these issues by regularization, a form of regression that imposes a penalty on the size of logistic regression coefficients, trying to shrink them towards zero (Ng, 2004; Stock et al., 2012). The Least Absolute Shrinkage and Selection Operator (lasso) or L-1 regularized lasso models and the L2 regularized ridge models allow for this approach. The L-1 regularizer in lasso models can force some of the coefficient estimates in the model to be exactly equal to zero, identifying features that are least important or least related to the outcome of interest. The L-2 regularizer in ridge models on the other hand shrinks the coefficient values towards zero, but never to an absolute zero, making it suitable for use when trying to identify coefficient values of variables that have some known potential relationships. We perform lasso prior to ridge for data reduction, where we use lasso to identify the features with a non-zero coefficient for inclusion in the ridge model. The ridge model then limits features to those determined by lasso to create the most optimal model for interpretation (Fig. 1.).

Lasso and Neural Networks. While the regularized regression models identify individual features of meaning, it is also helpful to know features occurring in combination that have meaning. Artificial neural networks are powerful statistical tools that can perform non-linear discriminant analyses and identify patterns of features that are associated with a given outcome of interest. In the past few years, we have seen their increased used in social sciences and epidemiology (Duh et al., 1998; Kreatsoulas & Subramanian, 2018; Qian & Sejnowski, 1988; Seligman et al., 2018). As with ridge models, neural networks involve a regularized approach to mitigate effects of overfitting and multi-collinearity. So again, we first use lasso findings for data reduction, and then use the reduced number of features to test with our neural networks models (see Fig. 1). We use feed forward neural networks, where the input travels in one direction; data passes through the input nodes and exits on output node. The neural network is a fully connected set of nodes organized into a number of layers, known as hidden layers. Nodes are logical structures composed of two parts; the first part receives incoming information (inputs) from possibly many sources, and the second part mathematically transforms the input into output information (outputs). The hidden layers define the successive linking of inputs and outputs resulting in a group of features while accounting for non-linear relationships. The neural network model used *tanh* function for transformation, which is mainly used in classification models. The model used batch normalization, with 100 epochs and a batch size of 100. The different weights for each input node was estimated iteratively using the training data set in such a way that the error function was minimized. The number of hidden layers was decided based on iterative estimation. A model with four hidden layers was chosen since it had the maximum performance in terms of balanced error rate and an indicator of accuracy - area under the receiver operating characteristic curve.

We provide further details of the three machine learning algorithms used in our study in Appendix A.

### Testing the performance of the logistic regression, ridge, and neural network models

To test the performance of our models, we assessed them for an indicator of accuracy and error. We calculated the area under the receiver operating characteristic curve (ROC AUC), which is used as an indicator of accuracy. The receiver operating characteristic (ROC) curve is a plot of the test true-positive rate (y-axis) against the corresponding false-positive rate (x-axis); i.e., sensitivity against specificity. ROC AUC, the area under the ROC curve, is often used to summarize test performance of models. Simplistically, AUC is the chance that a randomly selected observation (i.e., survey participant) who had a child marriage will be classified as that (Hanley & McNeil, 1982).

To test the error of our models, we estimated their balanced error rates (BER). BER is calculated as [1-0.5*(True Positive Rate (TPR) + True Negative Rate (TNR))]. The TPR and TNR were calculated by comparing the predicted and actual values in the test dataset. The machine learning models return probabilities of classification instead of dichotomous categories of 0 and 1 for each observation. We used 0.5 as the decision threshold for classification, which is the default used in many machine learning classification tasks (Chen et al., 2006). Based on this threshold and the predicted probabilities, observations in the test datasets were predicted to be positives or negatives. TPR is the ratio of true positives and overall actual positives, while TNR is the ratio of true negatives and overall actual negatives. BER is a reliable metric when assessing unbalanced datasets, with either large number of positive instances of the outcome, or large number of negative instances. For this analysis, since the prevalence rate of child marriage is less than 50%, BER offers robust estimates of the accuracy of the predictive models.

We estimated both ROC AUC and BER for the test dataset, applying this for each of our models separately; this approach supports indication of generalizability of our models to this dataset.

Final models for each model - traditional regression, ridge, neural networks - ranked features associated with child marriage in terms of “feature importance,” or coefficient values. We estimated these based on the value of a feature or risk factor when it was randomly permuted across the data. The difference in the mean squared error of the value from the random permutation and the actual value of the feature gives us the feature importance. This measures the “importance” of a feature in the sense that it captures the improvement in prediction error attributable to one feature, holding other features constant. We viewed meaningful features as those with a coefficient value higher than the knee point of the coefficient value graph. We used the mathematical definition of curvature for a continuous variable as the basis of the knee-point definition for our analysis (Satopaa et al., 2011). For any continuous function *f*, there is a standard closed-form that defines its curvature at any point as a function of its first and second derivative. And, the maximum curvature of this function indicates the levelling off effect. In other words, beyond the knee point, or the point of maximum curvature, the curve for the function becomes flat. In our analysis, the coefficient values of all the variables from the regression models were sorted from high to low. These coefficient values were then plotted and assessed to identify the point of maximum curvature (using *kneed* library in Python). The variables with coefficient values higher than the knee point, or the point where the coefficient curve becomes flat, were extracted as the meaningful features.

### Iterative categorization: a systematic approach to identifying predictive themes

The results from the ridge logistic regression (implemented after a lasso logistic regression model) presented a list of 36 features with a coefficient value higher than the knee point or point of maximum curvature of the coefficient curve. The goal of our study was to identify patterns or broad themes among the identified features, given the breadth of features and content areas covered by the survey. We thus followed a systematic approach which merged the hypothesis generating qualitative methodology of grounded theory and theme generation (Glaser & Strauss, 1999), with machine learning algorithms. Grounded theory involves an inductive analysis of text by domain experts (i.e., coders with content expertise) who review data to generate and code themes until no new themes are able generated (Glaser & Strauss, 1999). The process is iterative such that as more data are analyzed or become available, new themes may emerge, in which case all data would be re-analyzed for these new themes. Coders generate and agree upon themes together but code separately to allow for inter-coder reliability testing; any themes not agreed upon are discussed until agreement is reached or, if necessary, a third party domain expert decides. The final resultant themes are then used to generate theory and create testable hypotheses about a given phenomenon that was the broader focus in the text.

**Fig. 2:**
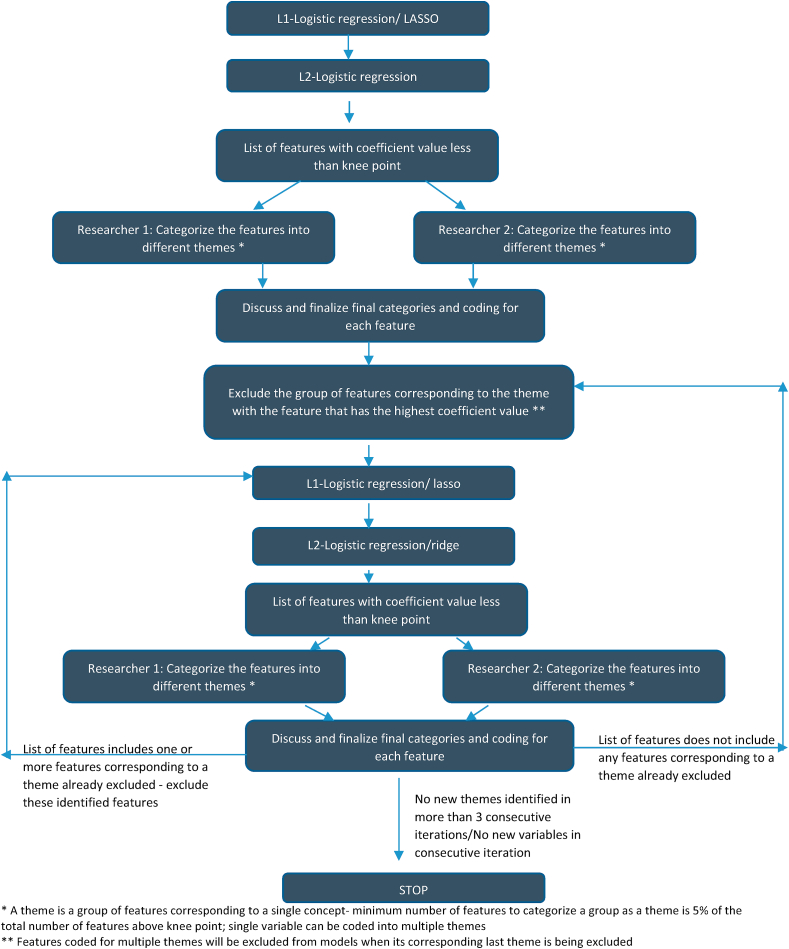
Flowchart for iterative categorization of predictor variables. * A theme is a group of features corresponding to a single concept-minimum number of features to categorize a group as a theme is 5% of the total number of features above knee point; single variable can be coded into multiple themes ** Features coded for multiple themes will be excluded from models when its corresponding last theme is being excluded.

Building from the grounded theory approach, we developed an inductive and iterative process of analyzing the features generated from the ridge logistic regression. Specifically, two coders with expertise and training on issues of child marriage in India (AR and ND) took the initial lasso and ridge logistic regression results and generated themes based on the resultant features. We developed themes based on features related to a unifying concept, if the number of features within a given theme were in number at least 5% of the total number of identified features above the knee point. We selected the arbitrary amount of 5% (~3 features) as a conservative estimate to allow identification of as many coherent themes as possible, while also restricting over-analysis of thematic data. We developed themes were to reflect distinct, specific, coherent and relevant dimensions, for example socio-economic positioning. A single feature could be included in multiple themes. If we found that a feature corresponded to multiple themes, we retained that feature in the next model iteration, removing it from an iteration only after we found it unconnected to any remaining themes in the model. Once we generated the themes and coded the features to those themes in this first model, we took the features attached to the theme that had the highest variance, or highest coefficient value, and dropped those features from the next iterative model. We then ran the next iterative model after eliminating these features, and we repeated our coding procedure with this second iterative model. As new features arose in each iterative model, we would generate new themes, if we found the number of new features related to a theme was sufficient to meet criteria for theme generation. If we found features from a prior theme in a new iteration of the model, we removed those features and ran the model the again. The process of cross-validation, and evaluation of the models on the test dataset was done after each round of this process of iterative categorization. We repeated this until we found no new emerging themes for at least three consecutive iterations, found no new variables in a given iteration, or when the ROC AUC was less than 75%. The decision around optimal value for ROC AUC statistic is subjective, based on the study context and subject (Ghaoui et al., 2010). An ROC AUC value of 50% is considered poor; our first iteration where all variables were included in the model had a ROC AUC of 91%. The initial design of splitting the dataset into train and test dataset was retained throughout this process of iterative categorization. The test dataset was used to estimate the ROC AUC. Once we completed this process, we listed the themes across our iterative models and examined them for theory development and hypothesis generation (See Fig. 2 for resultant themes.). It should be noted that consensus between coders was reached for all coded features across all models. This approach was developed specifically for this study and to test the utility of the application of this methodology to help clarify machine learning findings with content expert input for better interpretation of findings.

## Results

### Sample characteristics

This analysis included data from 78,542 ever-married women of age 20–24 years, around 41% of whom were married before the age of 18 (Table 1). Of those who had a child marriage, 75.2% married on or after turning 15 years of age, and 21.1% married at the age 10–14 years. This sub-sample of women who married before age 18 reported lower literacy, education and wealth, relative to the total sample; the subsample was also more representative of rural residents and residents of eastern India relative to that seen for all India.

**Table 1:** Sample characteristics.

	Total sample	Sub-sample of women who had a child marriage
	(N = 78,542)	(n = 31,066)
		40.9% of total sample
Characteristics	Wtd % (unwtd n)	Wtd % (unwtd n)
Literacy	76.7% (59,148)	67.7% (20,206)
Years of schooling		
No education	18.2% (15,408)	25.4% (8726)
Primary	13.5% (10,792)	18.1% (5594)
Secondary	56.3% (43,921)	52.8% (15,695)
Higher	12.1% (8421)	3.7% (1051)
Wealth index status:		
Poorest	19.6% (16,921)	26.8% (9188)
Poorer	22.7% (19,030)	27.1% (8821)
Middle	22.8% (17,460)	22.9% (6688)
Richer	20.6% (14,501)	16.1% (4328)
Richest	14.3% (10,630)	7.1% (2041)
Religion		
Muslim	15.2% (10,917)	15.4% (4337)
Hindu	80.7% (65,855)	81.2% (24,495)
Others	4.1% (1770)	3.4% (2234)
Caste		
SC/ST	32.2% (29,044)	34.8% (12,185)
OBC	45.6% (32,766)	44.3% (12,868)
Other castes/General caste	22.3% (16,732)	20.9% (6013)
Place of residence:		
Rural	72.5% (60,438)	77.9% (25,355)
Urban	27.5% (18,104)	22.1% (5711)
Region of residence		
North	13.1% (14,798)	11.7% (5045)
West	13.9% (6303)	13.6% (2510)
South	19.6% (8870)	17.0% (3049)
Northeast	3.3% (9034)	3.8% (3735)
East	25.6% (16,538)	31.4% (7939)
Central	24.5% (22,999)	22.6% (8788)

### Performance of different models and resultant predictors

**Fig. 3:**
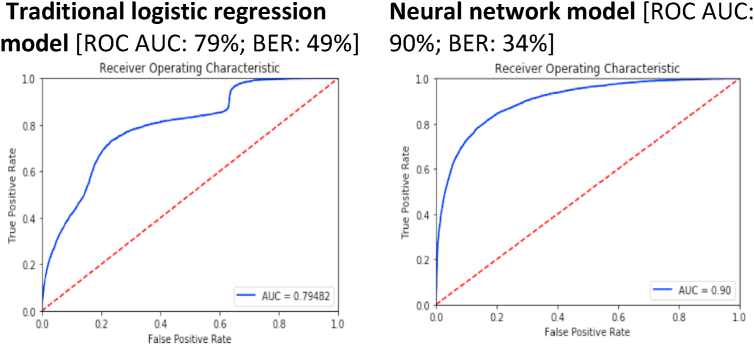

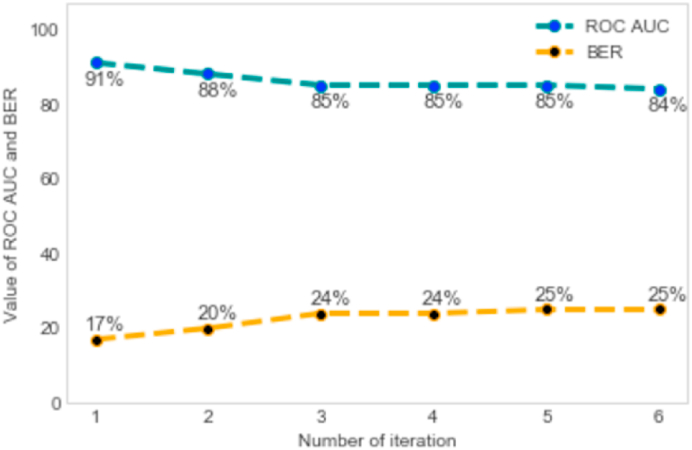
a: Receiver Operating Characteristics Area Under Curve (ROC AUC) and Balanced Error Rate (BER) for traditional logistic and neural network models. b: Receiver Operating Characteristics Area Under Curve (ROC AUC) and Balanced Error Rate (BER) for different iterations of lasso with ridge logistic regression model.

The traditional logistic regression model demonstrated the low accuracy and performance, with a ROC AUC (accuracy) of 79% and a BER (balance/performance) of 50% (Fig. 3). The ridge regression model, in contrast, had a ROC AUC of 91% and a BER of 16%, and the neural network model – with four hidden layers - had a ROC AUC of 90% and a BER of 34%. Generated predictors differed across models (Table 2.). The traditional model's predictors largely focused on contraceptive use, with users of various types of contraceptives more likely to report child marriage. Predictors from the ridge logistic regression model included indicators of longer marital duration/cohabitation, younger age at first sex, motherhood and higher fertility, geography (Indian states, e.g., Rajasthan, Jharkhand, Assam), and poverty. Neural network predictors also included many predictors seen in the ridge logistic regression models, but some differences, such as inclusion of different states (e.g., Uttar Pradesh) and backward caste.

**Table 2:** Identified predictors from the three models: traditional logistic regression model, ridge logistic regression model, and neural network model.

Traditional logistic regression model^a^	Ridge Logistic Regression Model^a^	Neural Network^a^
Reason for not currently using any contraceptive method: not having sex	Age at first sex: 15–17 years	Marriage to first birth interval (months): 25+ months
Type of mosquito bed nets slept under last night: treated nets	Marriage to first birth interval: 25–609 months	Cohabitation duration: 0–4 years
Have used IUD	Cohabitation duration: 10–14 years	Age at first sex: 15–17 years
Have used lactational amenorrhea method	State: Rajasthan	Cohabitation duration 5–9 years
Marriage to first birth interval: negative (had a child before marriage)	Years lived in place of residence: 6–94 months	Marriage to first birth interval: 0–12 months
Have used rhythm method	Birth between last and interview: no	Age at first sex: at first union
Have used abstinence method	Second child is alive: yes	Years lived in place of residence: less than one year
Time since last menstrual period: more than a month	First child is twin: no, single birth	State: Rajasthan
Knows at least one modern method	First child is alive: yes	Births in past year: one
Have used the pill	Age at first sex: 7–14 year	Respondent's mother tongue: Hindi
Have used sterilization	State: Jharkhand	Second child: single birth
Have used other modern methods	Live birth between births: no	First child: single birth
	Second child is twin: no, single birth	Years lived in place of residence: 1–5 years
	First child lives with whom: respondent	District: Chandauli (Uttar Pradesh)
	Cohabitation duration: 15–19 months	First child is alive
	Third child is twin: no, single birth	District: Siddharth Nagar (Uttar Pradesh)
	Live birth between births: no	Gave child any fruits in the past 24 h: no
	Third child is alive: yes	District: Shrawasti (Uttar Pradesh)
	Highest educational level: secondary	Belongs to other backward class
	Births in past year: no births	
	Sex of first child: female	
	Unmet need for contraception: infecund, menopausal	
	Pregnancies terminated before calendar beginning: yes	
	Third child lives with whom: respondent	
	Second child lives with whom: respondent	
	Sex of first child: male	
	Sex of second child: female	
	No unmet need	
	Second child lives with whom: lives elsewhere	
	Sex of third child: male	
	Sex of second child: male	
	Wealth index: poorest	
	State: Arunachal Pradesh	
	Children under 5 slept under mosquito bed net last night: yes, some	
	Marital duration at sterilization: 5–9 years	
	State: Assam	

a Predictors are those features that are above the knee point in coefficient curve; predictors presented in order of coefficient size (largest to smallest).

### Iterative categorization and theme generation

Our iterative categorization exercise began with theme generation based on the initial ridge regression model, from which we identified six themes: sexual history, marriage, reproductive history and fertility, geography, social and economic status, and family planning. Subsequently, each iterative model dropped features related to the theme that had the highest value of coefficient (i.e., contributed the most variance to our outcome). Thus, round two dropped the theme ‘sexual history,’ and this second model yielded new features resulting in a new theme: ‘receipt of benefits from health systems.’ Round three dropped the ‘reproductive history and fertility’ theme and generated no new themes. Round four dropped the ‘marriage’ theme and generated no new themes. Round five dropped the ‘geography’ theme and generated two new themes: exposure to media, and diet. Round six dropped the ‘socio-economic status’ theme and generated no new themes. We did not identify any new features to code during the seventh round of analysis, so we stopped the iterative exercise. This procedure resulted in generation of nine explanatory themes related to our child marriage outcome (See Table 3 for the list of features corresponding to each of the nine resultant themes. All features are positively correlated with child marriage.). It also demonstrated the themes that contributed the most variance to our outcome of interest (See Supplemental Table A for the results by iteration.).

**Table 3:** Identified predictors for corresponding themes from the iterative categorization exercise.

Sexual history	Marriage	Reproductive history and fertility	Geography	Socio-economic status	Family planning	Receipt of benefits from health systems	Exposure to media	Diet
Age at first sex: 15:17 years	Cohabitation duration 5–9 years	Ever had a terminated pregnancy: yes	State: Rajasthan	Wealth index: poorest	Have used a method	During pregnancy with second child, received benefits from Anganwadi/ICDS centre: no	Frequency of reading newspaper or magazine: not at all	Frequency eats fruits: occasionally
Age at first sex: 7:14 years	Cohabitation duration 10–14 years	First child- single birth	State: Arunachal Pradesh	Wealth index: poorer	Marital duration at sterilization: 5–9 years	First child received benefits from Anganwadi/ICDS centre, last 12 months: no	Frequency of listening to radio: less than once a week	Frequency eats eggs: never
	Cohabitation duration 15–19 years	Second child-single birth	State: Jharkhand	Wealth index - rural: poorest	Ever used anything or tried to delay or avoid getting pregnant: yes	While breastfeeding first child, received benefits from Anganwadi/ICDS centre: no		
	Number of unions: more than once	Third child - single birth	State: West Bengal	Wealth index - rural: poorer	Reason of last discontinuation: wanted to become pregnant	During pregnancy with second chid, received benefits from Anganwadi/ICDS centre: no		
		First child is alive	State: Assam	Literacy: cannot read at all	Ideal number of children: 3+	While breastfeeding second child, received benefits from Anganwadi/ICDS centre: no		
		Second child is alive	District: Mandsaur (Madhya Pradesh)	Highest educational level: no education	Unmet need: no unmet need since woman is infecund	Child received benefits from Anganwadi/ICDS centre, last 12 months: no		
		Third child is alive	District: Ajmer (Rajasthan)	Highest educational level: primary	Is infecund	Ultrasound test: no		
		First child lives with whom: respondent	District: Siddharth Nagar (Uttar Pradesh)	Highest educational level: secondary		Ultrasound at any pregnancy: no		
		Second child lives with whom: respondent	District: Shrawasti (Uttar Pradesh)	Religion: Hindu		Benefits received while breastfeeding first child: health and nutrition education: no		
		Third child lives with whom: respondent				Frequency first child had weight measured by Anganwadi/ICDS centre, last 12 months: less often		
		No births in past year				Ultrasound test for second pregnancy: no		
		Sex of second child: female						
		Sex of first child: female						
		Sex of first child: male						
		Sex of second child: male						
		Sex of third child: female						
		Sex of third child: male						
		Marriage to first birth interval (months): 25+ months						

## Conclusion

We conducted this study to apply machine learning technologies to understand child marriage in India, with the goal of generating previously unrecognized predictors (or correlates, as these are cross-sectional data and causality cannot be presumed) of this outcome for purposes of intervention targeting. We also sought to build our theory of understanding on this issue and to generate new hypotheses for exploration and focus to accelerate elimination of the practice in India. Findings from this work demonstrate three important points. First, machine learning methodologies in the forms of lasso regression and neural networks appear to be valid for detecting important correlates of social health outcomes such as child marriage, with the regularized regression and neural network models performing better than the use of traditional regression in machine learning. Second, building on prior research demonstrating the importance of socioeconomic, geographic and fertility-related correlates of child marriage, current findings demonstrate that poor integration into the health and social welfare systems and inadequate diet are key correlates of child marriage that may explain prior research documenting associations between child marriage and poorer maternal and child health outcomes. Finally, our novel expansion of machine learning methods to include an iterative approach to models for theme generation, analogous to grounded theory approaches used in qualitative research, appears to be highly instrumental in generating themes and variables that may otherwise be hidden from our understanding. This approach expands capacity for thematic analysis for hypothesis generation through machine learning models, but at the same time demonstrates the importance of having experts in both content/domain expertise as well as machine learning methodologic expertise to undertake this work. Machine learning methodologies as applied to public health and social and behavioral sciences can only be useful with proper preparation of the database in terms of variable selection and content analysis of variables generated when these content/domain experts are included in the process.

A comparison of the features or variables generated from the three type of models - traditional regression, the regularized models -lasso and ridge regression, and neural networks – yield somewhat different findings. The traditional model showed poorer performance and accuracy than did the ridge and neural network models, suggesting that the latter two models are more robust in identification of predictors. The traditional regression model also yielded, for the most part, only contraceptive use-related variables as predictors, including both modern methods (e.g, pill, IUD, sterilization) and traditional methods (e.g., rhythm, abstinence). Within our sample of married 20–24 year olds, these findings may indicate that those who married prior to 18 may simply have be more likely to have engaged in contraceptive use because of greater opportunity (i.e., due to longer duration of marriage) and potentially completion of desired fertility. Prior research from India on this issue has demonstrated that, among 20–24 year old, those who marry before 18 are more likely to report sterilization (Raj et al., 2009), the most common form of contraceptive used in India (IIPS, 2018; Raj et al., 2009). The regularized regression and neural network models, in contrast, generated a far greater array of predictors. These included features indicative of child marriage and demonstrated as correlates of the practice in prior research, including the national demographic report on these data (IIPS, 2018; Raj, 2010). These include longer duration of marriage, younger age at first sex, any children and higher fertility, socioeconomic vulnerability (poverty and lower caste), and specific geographies (e.g., state of Rajasthan, and in the neural network, specific districts of Uttar Pradesh). These findings demonstrate the validity of this approach as compared with findings seen from prior research, but the findings offer little in the way of generating new, unanticipated variables that could expand our theory of understanding.

In an effort to generate a broader array of novel themes from machine learning than the standard machine learning approaches would allow, we created a theme generation exercise with ridge regression, our highest performing model, where we conducted iterative models to illuminate themes that might otherwise be hidden by those that account for high variance in our outcome. This approach borrowed from grounded theory approaches used in qualitative research (Glaser & Strauss, 1999) to illuminate findings via thematic analysis of model-generated features (i.e., variables) conducted by content/domain experts. The order of removal of themes highlights those themes that most contribute to variance; sex (earlier age at first sex), marriage (primarily features of marital duration), fertility (any and more children), geography (e.g., Rajasthan, West Bengal, districts in Uttar Pradesh), socio-economic status (wealth, rural residence, low education and literacy), and family planning (use of various contraceptives). These were all themes generated in the first model, though with more features revealed via the iterative process. These themes have also been documented in prior research on this topic and recognized by relevant national and international bodies (Raj, 2010; UNICEF, 2018; UNGA, 2018; IIPS, 2018). Importantly, upon removal of themes, new themes were identified that heretofore have received less attention related to the topic of child marriage. These were non-receipt of benefits from health systems, no exposure to media, and poorer diet, in that order regarding contribution to variance.

Consideration of these less recognized themes offer important insights into potential interventions, and may help explain previously identified findings on poor maternal and child outcomes related to child marriage. Findings related to non-receipt of health benefits largely were based on non-receipt of services from local nutrition centers, known as Anganwadi or Integrated Child Development Services (ICDS) centers, a striking finding given that these are programs specifically available for the rural poor, where child marriage is more likely. Lack of connection to and receipt of these services may help explain prior research demonstrating poorer child nutrition outcomes among children of women married as minors in India, and elsewhere (Efevbera et al., 2017; Raj et al., 2010a). The association between low mass media exposure and child marriage has been shown in prior research (Gage, 2013; Rumble et al., 2018; Singh et al., 2012), and may be indicative of low access but also, in the case of newspaper readership, low literacy. Poorer diet as indicated by lack of egg intake and only moderate fruit intake has received little discussion in the literature. Lack of egg intake may be a product of religious doctrine from Hinduism; being Hindu was associated with child marriage in our findings. Moderate rather than higher fruit intake may be indicative of poverty or geography. Regardless, these features may indicate need for greater nutritional focus for this population, findings reinforced by the above noted lack of engagement with benefits from Anganwadi Centers. Overall, these findings extend prior research by documenting that women who marry as minors are receiving less reach from public health and welfare efforts, and possibly explaining the mechanisms through which child marriage is associated with poorer maternal and child nutritional health outcomes, as shown in prior research from India (Goli et al., 2015; Raj et al., 2010a). Findings also demonstrate the need for more focused nutritional intervention for this group, inclusive of improvements in outreach and engagement from Anganwadi Centers. Perhaps most importantly with regard to the value of methods, these findings also illustrate the value of this thematic generation approach with iterative models and the importance of ensuring teams undertaking such analyses include both experts in content and in machine learning methodologies.

While findings from this study offer important insights into both advancements in methodologies for application of machine learning in public health and for understanding child marriage in India, we must consider them in light of certain study limitations. First, while the three models used in this study perform well and provide meaningful results, there are multiple machine learning algorithms available. It is possible that few of the other existing methods outperform our methods, and future research could consider testing other models for comparative analysis. To further explore potential interactions between variables, we also tested a neural network model without lasso for feature selection and a random forest model, a popular machine learning algorithm that has also been used in epidemiologic research (Kanerva et al., 2018). These models demonstrated higher balanced error rates and lower accuracy rates than our original models, validating the robustness of our approach. Next, while the survey data used in this analysis was quite comprehensive in demographic data it was not comprehensive across all domains that might be meaningful to the understanding of child marriage. Hence, under-representation of certain types of features and thus potential themes may be a concern; for example, economic indicators are less comprehensively assessed in these data and may have become its own theme had more diverse features on this theme been included in the data set. Additionally, measurement error may be more problematic in big data analyses due to inadequate information about potential data artifacts from this data set (Mooney & Pejaver, 2018a). This concern is somewhat alleviated by inclusion of co-authors on this study involved with the original collection of these data. With regard to neural networks, we cannot necessarily know how any given prediction was made or the relative value of a given individual feature since the layers are hidden and the output is for the network of features (Eck, 2018). The ridge models however provided this insight for out consideration, suggesting the value of including both approaches when using machine learning.

Additional limitations relate to the reliance on survey data obtained through self-report, which are subject to both recall bias and social desirability bias, as well as to the limited generalizability of study findings to India. Our data are cross-sectional, as noted previously, so causality cannot be assumed; identified “predictors” may have preceded or followed marriage. Finally, it must be acknowledged that this approach to data analysis does not mean that there are not other features or variables in the data set that are associated with child marriage. Prior research documents marital violence for example to be associated with this outcome (Kidman, 2017; Raj, 2010; Raj et al., 2010b). These findings only reflect the themes from features that account for the most variance in the outcome. Further, they can only account for features available in this dataset.

Summary of Conclusion and Implications. Findings from this study demonstrate the utility and validity of machine learning application for understanding social health issues such as child marriage, but with some caveats. There is greater value to regularized logistic and neural network applications relative to traditional logistic regression with machine learning for accuracy and balance of the model, with a greater number of features and variables generated. However, for well-researched phenomena such as child marriage, novel variables are not easily identified via this procedure. To a great extent, the variables or features accounting for the greatest variance in the outcome are those that would otherwise be expected such as duration of marriage, fertility, and socio-economic marginalization. For these reasons, an iterative modeling approach with theme generation, as developed for this paper, offers important advantages over the standard machine learning approaches by providing greater opportunity for theme generation and identification of themes and features potentially hidden by features or variables too strongly associated with the outcome of interest. Content expert analysis of machine learning output via qualitative coding of variable output and thematic generation can offer greater clarity for interpretation while also maximizing the utility of machine learning methodologies to generate new hypotheses.

This approach offers important insights into the issue of child marriage for India not captured in prior research. Specifically, we find non-utilization of health system benefits related to nutrition for mothers and infants associated with child marriage, a finding that helps explain previous research documenting poorer nutritional health outcomes for mothers and children affected by child marriage in India (Goli et al., 2015; Raj et al., 2010a) and elsewhere (Efevbera et al., 2017, 2019). Such findings also correspond to prior intervention research from India documenting that public health system interventions may have poorer reach and impact on those married as children (McDougal et al., 2017). They additionally expand our theory of child marriage to show inadequate connection to public health systems of care, which can be explored via generation and testing of new hypotheses related to this theory. Hence, these machine learning methodologies, particularly when enhanced with an iterative modeling and theme generation approach with a combination of content and methods experts, offer important insights into child marriage in India that can be expanded to other nations and other public health-relevant social and behavioral science phenomena as well.

Certainly, more research is needed regarding barriers to public health program use among married adolescents and young women is needed to understand and counteract these accordingly. However, public health impacts cannot simply await more research. Public health and nutrition efforts must prioritize resources and home-based outreach in geographic areas known to have ongoing higher rates of child marriage and in those with historic high rates of child marriage, as nutritional deficits may be continuing in these contexts. Nutritional programs for adolescent girls should also be prioritized, which would be a benefit for these girls into adulthood and for their offspring should they bear children, regardless of age at marriage and childbirth. Importantly, prevention of child marriage is also key to address this concern, and may be all the more important at this time, as the COVID-19 pandemic and related economic impacts continue to escalate in India and have been linked to an increase in the practice of child marriage in the multiple states.

## Author contributions

Anita Raj led conceptualization of this paper, led development and supported implementation of the iterative theme generation process, drafted the introduction and conclusion of the paper, and provided substantive revisions and finalized the complete paper.

Nabamallika Dehingia conducted all analyses for this paper under the direction of Julian McAuley, Anita Raj, and Lotus McDougal. She drafted the methods and results for the paper, and provided substantive contribution to the development of all analyses for this work including development of the novel approach to iterative theme generation. She provided substantive revisions to the full paper and approved the final submitted paper.

Abhishek Singh was involved in generation of data for this paper, guided data pre-processing plans and variable selection, provided substantive input and reviews of the paper including recommendations for future research and public health programs in India. He approved the final submitted paper.

Lotus McDougal supported conceptualization of the paper, led data pre-processing and variable selection, supported all data analysis plans and reviewed all analyses, supported drafting of the methods of the paper, provided substantive review and editing to the full paper, and approved the final submitted paper.

Julian McAuley led design of machine learning analyses, guided all machine learning analyses conducted by Nabamallika Dehinigia, provided substantive reviews and inputs into the writing of the paper, and approved the final submitted paper.

## Role of the funding source

This study was funded under a grant from the 10.13039/100000865Bill and Melinda Gates Foundation (Grant number OPP1179208; PI: Anita Raj). The funding source had no involvement in this research or preparation of this article in any way.

## Declaration of competing interest

None.
