# Supplementary material for: Application of machine learning to understand child marriage in India
Source: SSM Popul Health. 2020 Dec 5;12:100687. doi: 10.1016/j.ssmph.2020.100687 (PMC7732880; doi:10.1016/j.ssmph.2020.100687)
Supplement: Multimedia component 2 [file mmc2.docx]

**Appendix A: Machine Learning Algorithms**

Traditional Regression. Traditional logistic regression with a binary response is denoted by the equation:

$$P\left( y=1 \right)= \frac{1}{1+ e^{-\theta\beta}}$$

We obtain regression coefficients by maximizing the log-likelihood function:

$$l_{\theta}\left( y | X \right)= \sum_{i} -log(1+ e^{{-X}_{i}\theta})+ \sum_{y_{i}=0} -X_{i}\theta$$

Where X is the vector of features or variables and θ is the column vector of the regression coefficients. However, in cases involving high dimensionality, or large number of features, the ordinary logistic regression has a few problems: multicollinearity, and over-fitting, and computational difficulties.

Lasso. To address concerns posed by the presence of large number of features in a dataset is regularization. Regularization is a form of regression that imposes a penalty on the size of logistic regression coefficients, trying to shrink them towards zero. Regularized estimators are thus restricted maximum likelihood estimators (MLE), since they maximize the likelihood function subject to restrictions on the logistic regression parameters.

To develop parsimonious ridge and neural network models, with fewer and more relevant features, we used lasso. Lasso uses an L-1 penality for both variable selection and shrinkage, and when the λ is sufficiently large, it can force some of the coefficient estimates to be exactly equal to zero, giving us models with lesser number of predictors/features. The log-likelihood function for lasso takes the form:

$$l_{\theta}\left( y | X \right)= \sum_{i} -log(1+ e^{{-X}_{i}\theta})+ \sum_{y_{i}=0} -X_{i}\theta- \lambda|\theta|$$

The difference between the traditional regression and lasso is the last term: $\boldsymbol{\lambda|\theta|}$**.** This term is the regularizer and is used to optimize the log-likelihood function. Simply put, this allows the model to carry out multiple iterations for the log-likelihood function to find the best values for all the betas (coefficients) in the equation, while mitigating overfitting and bias.

Ridge. Ridge or L-2 regularized logistic regression is also obtained by maximizing the log-likelihood function with a penalized parameter applied to all the coefficients except the intercept, resulting in the following constrained maximization equation:

$$l_{\theta}\left( y | X \right)= \sum_{i} -log(1+ e^{{-X}_{i}\theta})+ \sum_{y_{i}=0} -X_{i}\theta- \lambda{|\left| \theta\right||}_{2}^{2}$$

Where λ is the tuning parameter for the L-2 regularized model. The larger the λ, the stronger its influence is, and the smaller are the parameter estimates. When λ = 0 the solution is the ordinary MLE, whereas if λ→∞, the θ all tend to 0. Different approaches to choose the value of λ have been described in existing literature. The tuning parameter for ridge, λ, was selected using k-fold cross validation, using the method described above for lasso.

Neural network. We used feed forward neural networks, where the input travels in one direction; data passes through the input nodes and exits on output node. The neural network is a fully connected set of nodes organized into a number of layers, as noted in the text of the document. Nodes are logical structures composed of two parts; the first part receives incoming information (inputs) from possibly many sources, and the second part mathematically transforms the input into output information (outputs). For any particular problem, the numbers of input and output units are fixed. In the current analysis, all the independent features form the output, and the output is binary variable child marriage. Between the input and output nodes, are the hidden layers. The layers define the successive linking of inputs and outputs. The higher the number of hidden units, the higher is the functional complexity of the equation relating input to outcome. Thus, higher order, non-linear associations between the input units and the output can be modelled. We calculated the value of each hidden unit by summing the product of the input units with their associated weights (strength of the relationship for the respective link) and applying a non-linear activation function to this summation. The activation functions are simple transformations. In this study, the output required a binomial response, so we used *tanh* function to perform this transformation. *Tanh* is mainly used for classification algorithms.

$$\tanh\left( z \right)= \frac{e^{z}- e^{-z}}{e^{z}+ e^{-z}}$$

The effect of this transformation was to map values of z, which can range from −∞ to +∞ , into the narrower range of −1 to +1 . For the computation in the output layer, the algorithm was similar to that in hidden layers, except that we used only one output node as we only have one output of interest, child marriage. We estimated the different weights for each input node iteratively using the training data set in such a way that the error function was minimized.
